# Effects of Aquatic Physiotherapy on Pain and Sleep Quality in Women With Fibromyalgia: A Pre‐Post Study

**DOI:** 10.1002/pri.70150

**Published:** 2025-12-19

**Authors:** Josilene Câmara Ferreira, Maria Madalena Corrêa Melo, Raylson De Jesus Pereira Barros, Luana Rodrigues de Lima Sipaúba

**Affiliations:** ^1^ Department of Physiotherapy Santa Terezinha University Center São Luís Maranhão Brazil; ^2^ Department of Pharmaceutical Sciences University of São Paulo São Paulo Brazil

**Keywords:** aquatic physiotherapy, fibromyalgia, pain, sleep quality

## Abstract

**Background and Purpose:**

Fibromyalgia (FM) is a chronic syndrome characterized by widespread pain, fatigue, sleep disturbances, and reduced quality of life. Non‐pharmacological interventions, such as aquatic physiotherapy (AP), have been proposed to alleviate symptoms and improve both physical and psychological outcomes. Therefore, this study aimed to evaluate the effects of an 8‐week AP program on pain intensity and sleep quality in women with FM, and to explore changes in specific sleep dimensions, including latency, duration, efficiency, disturbances, and daytime dysfunction.

**Methods:**

This was a quasi‐experimental pre‐post study including thirteen women with FM who participated in an 8‐week AP program at a school clinic. The program consisted of two 45‐min sessions per week in a therapeutic pool heated to 33°C–35°C, including warm‐up, aerobic and resistance exercises, and relaxation. Pain was assessed using the Visual Analogue Scale (VAS), and sleep quality using the Pittsburgh Sleep Quality Index (PSQI). Measurements were taken before and after the intervention. Data were analyzed to examine within‐group changes rather than causal effects.

**Results:**

AP led to significant reductions in pain intensity, from severe to mild‐to‐moderate (VAS), and improvements in overall sleep quality (PSQI score reduced from 16 to 9), including enhanced sleep latency, duration, efficiency, disturbances, and reduced daytime dysfunction. Participants also reported improvements in functional capacity and psychological well‐being. No adverse events related to the intervention were observed.

**Discussion:**

Participation in the AP program was associated with reductions in pain and improvements in sleep quality among women with FM. These findings suggest potential benefits linked to the physical and psychosocial effects of aquatic exercise. Despite limitations such as the small size and absence of a control group, AP appears to be a feasible complementary intervention in public health and clinical settings.

## 1 Introduction

Fibromyalgia (FM) is a chronic, non‐inflammatory pain syndrome characterized by widespread musculoskeletal pain associated with symptoms such as sleep disturbances, fatigue, muscle stiffness, headache, constipation, anxiety, and depression (Bhargava and Goldin 2025). Globally, FM affects between 2% and 6% of the population, predominantly women, leading to substantial personal and socioeconomic burden (Marques et al. 2017; Mork and Nilsen 2012).

Chronic pain is the most reported symptom, often exacerbated by stress, climate changes, or physical exertion, and frequently localized at specific painful sites known as tender points (Bhargava and Goldin 2025; Siracusa et al. 2021). The syndrome is strongly associated with sleep disturbances, affecting 76%–90% of patients. These disturbances include difficulty in sleep induction, frequent awakenings, and non‐restorative sleep, which contribute to morning fatigue, cognitive deficits, and increased susceptibility to psychiatric disorders (Bigatti et al. 2008; Dauvilliers et al. 2001).

Management of FM requires a multidisciplinary approach that combines pharmacological and non‐pharmacological strategies (Burckhardt 2006). Among the latter, aquatic physiotherapy (AP) stands out as an effective intervention. The physical properties of water, including buoyancy, viscosity, hydrostatic pressure, and thermal conductivity, facilitate a greater range of motion and reduce the mechanical stress on joints, allowing patients to perform exercises that would be difficult or painful on land (Rodríguez‐Huguet et al. 2024; Zamunér et al. 2019).

In addition to alleviating musculoskeletal pain and fatigue, AP can improve muscle strength, joint flexibility, balance, and overall cardiovascular and respiratory function. Warm water immersion promotes vasodilation, increases blood flow, and enhances the removal of metabolic waste products, contributing to analgesic and anti‐inflammatory effects (Zamunér et al. 2019; Bidonde et al. 2014). Furthermore, the aquatic environment provides sensory stimulation that can compete with pain signals, leading to reduced perception of discomfort. Psychological benefits have also been reported, including decreased levels of anxiety and depression and improved overall well‐being, which are particularly relevant given the high prevalence of mood disturbances in patients with FM (Bhargava and Goldin 2025; Munipalli et al. 2024). These combined physical and psychological effects make AP a valuable component of a comprehensive management plan for individuals with FM.

Despite growing evidence supporting AP, most studies have focused on its effects in controlled or specialized settings, with limited data on its real‐world feasibility and impact in community or academic clinical environments. Addressing this gap is crucial to guide physiotherapy practice and public health programs aiming to expand access to effective, low‐cost interventions for FM management. In this context, the aim of this study was to evaluate the effects of an AP program on pain intensity and sleep quality in women with FM, including specific sleep dimensions such as latency, duration, efficiency, disturbances, and daytime dysfunction.

## 2 Methods

### 2.1 Study Design and Setting

This was a quasi‐experimental pre‐post study without a control group, conducted at the Santa Edwiges School Clinic, located on São Luís, Maranhão, Brazil, from March to April 2025. The intervention consisted of an 8‐week AP program, with two 45‐min sessions per week, conducted in a pool heated to 33°C–35°C. Each session included approximately 10 min of warm‐up and stretching, 25 min of aerobic and resistance exercises adapted to water buoyancy, and 10 min of relaxation. All sessions were supervised by a licensed physiotherapist specialized in AP. Potential confounding variables such as age, body mass index, and medication use were recorded at baseline.

### 2.2 Participants

The sample comprised 13 women with a medically confirmed diagnosis of FM attending AP at the School Clinic. Inclusion criteria were: women aged ≥ 18 years, confirmed diagnosis of FM, undergoing AP, signed informed consent, and at least 1 month of ongoing treatment. Participants with cognitive impairment affecting the understanding of study procedures were excluded.

### 2.3 Data Collection

The following instruments were used:

- Data collection form adapted from the physiotherapy assessment sheet of the Santa Edwiges School Clinic, including: personal data, comorbidities, medications, admission date, pain intensity, tender point locations, type of pain, other symptoms, clinical procedures, and number of sessions;
- Physiotherapy assessment sheet from the School Clinic;
- Pittsburgh Sleep Quality Index (PSQI), validated in Portuguese by Bertolazi et al. (2011), assesses seven components of sleep: subjective quality, latency, duration, efficiency, disturbances, use of medication, and daytime dysfunction. Scores > 5 indicate poor sleep quality;
- Visual Analog Scale (VAS) for unidimensional assessment of pain intensity (0 = no pain; 10 = worst imaginable pain), categorized as mild (Bhargava and Goldin 2025; Marques et al. 2017; Mork and Nilsen 2012), moderate (Siracusa et al. 2021; Bigatti et al. 2008; Dauvilliers et al. 2001), and severe (Burckhardt 2006; Rodríguez‐Huguet et al. 2024; Zamunér et al. 2019; Bidonde et al. 2014).

Data were collected by the researcher before and after the AP intervention. When necessary, additional information was retrieved from patient records to ensure completeness.

### 2.4 Statistical Analysis

Data were entered into Microsoft Excel (version 2019) and analyzed using SPSS (version 26). Categorical variables were presented as absolute (*n*) and relative (%) frequencies, while continuous variables were reported as mean ± standard deviation and median (minimum–maximum). Data normality was tested using the Shapiro–Wilk test. The Wilcoxon signed‐rank test was used to compare pre‐ and post‐intervention measurements, with a significance level of *p* < 0.05.

### 2.5 Ethics

The study protocol was approved by the institution's Ethics Committee following the Brazilian National Health Council Resolutions (National Health Council 2012) under number: 85200524.2.0000.8907, ensuring participant confidentiality and protection of personal data.

## 3 Results

### 3.1 Participant Flow

The study sample consisted of 13 women with a medically confirmed diagnosis of FM, treated in the AP sector of the Santa Edwiges School Clinic in São Luís, Maranhão, Brazil, between March and April 2025. All participants completed the intervention and were included in the final analysis with no dropouts.

### 3.2 Baseline Characteristics

The sociodemographic characteristics of the participants are shown in Table 1. The mean age was 51.5 ± 5.7 years. Most participants were single (46.2%) and had a monthly family income between 1 and 3 minimum wages (69.2%). Regarding occupation, 30.8% were homemakers and 30.8% were employed, while 46.1% had completed high school.

**TABLE 1:** Sociodemographic characteristics of women with FM undergoing aquatic physiotherapy.

Variables	*N* (%)
Age (years), Md ± SD	51.5 ± 5.7
Marital status	
Single	6 (46.2)
Married	5 (38.4)
Divorced	1 (7.7)
Widowed	1 (7.7)
Family income (minimum wages)	
Up to 1	3 (23.1)
1–3	9 (69.2)
3–5	1 (7.7)
Occupation	
Unemployed	1 (7.7)
Homemaker	4 (30.8)
Self‐employed	3 (23.0)
Employed	4 (30.8)
Retired	1 (7.7)
Education	
Incomplete elementary	1 (7.7)
Complete elementary	3 (23.1)
Complete high school	6 (46.1)
Complete higher education	3 (23.1)

### 3.3 Pain and Sleep Quality Assessment

Pain and sleep quality results are detailed in Table 2. The VAS scale score for pain decreased significantly from a median of 10 (range 8–10) to 5 (range 2–7) after the intervention (*p* = 0.001), representing a 50% reduction in pain perception.

**TABLE 2:** Pain intensity and sleep quality before and after aquatic physiotherapy intervention.

Scales/Components	Pre (Md, min–max)	Post (Md, min–max)	*p* ¥	% change
VAS	10 (8–10)	5 (2–7)	0.001	↓50%
PSQI total	16 (11–21)	9 (4–21)	0.002	↓44%
Subjective sleep quality	3 (1–3)	1 (0–3)	0.004	↓67%
Sleep latency	3 (2–3)	1 (0–3)	0.009	↓67%
Sleep duration	3 (1–3)	1 (0–3)	0.006	↓67%
Habitual sleep efficiency	3 (1–3)	1 (0–3)	0.007	↓67%
Sleep disturbances	2 (2–3)	2 (1–3)	0.014	↓33%
Use of sleep medication	3 (0–3)	0 (0–3)	0.317	↓100%
Daytime dysfunction	2 (0–3)	1 (0–3)	0.053	↓50%

Abbreviations: ¥, Wilcoxon test; PSQI, Pittsburgh sleep quality index; VAS, visual analog scale.

Global sleep quality assessed with the PSQI also improved significantly, decreasing from 16 (range 11–21) to 9 (range 4–21) (*p* = 0.002), corresponding to an approximately 44% improvement. PSQI component analysis showed significant improvements in subjective sleep quality, sleep latency, duration, habitual efficiency, and sleep disturbances (*p* < 0.05). Use of sleep medication and daytime dysfunction decreased but were not statistically significant (*p* > 0.05).

### 3.4 Summary of Results

Overall, AP produced substantial reductions in pain intensity and significant improvements in sleep quality, highlighting its role as an effective complementary intervention for managing FM. The largest improvements were observed in pain perception and subjective sleep quality.

## 4 Discussion

The present study aimed to investigate the effects of AP on pain and sleep quality in women with FM treated in a school clinic setting. The mean age of participants was 51.5 years, consistent with previous studies showing that FM mainly affects middle‐aged women, with relevant socioeconomic consequences (Oliveira et al. 2024; Salaffi et al. 2021). Although different interventions have been proposed for FM, few have compared their effects on multiple outcomes. A randomized clinical trial (RTC) (Fonseca et al. 2021) comparing AP and a health education program (HEP) found improvements in both groups, with HEP showing greater benefits in daily life impact. These findings suggest that AP is effective, but combining educational and physical approaches may provide more comprehensive and lasting benefits.

Regarding relationship status, a higher prevalence of single participants was observed (46.2%), differing from other studies reporting a predominance of married women (Oliveira et al. 2024; Freitas et al. 2017). The absence of a partner may influence pain perception, depression, anxiety, and other psychosocial symptoms associated with FM. Evidence from a Finnish cohort study (Grundström et al. 2021) suggests that being single, compared to being married, is associated with poorer mental well‐being and higher depressive symptoms throughout adulthood, particularly in women at certain life stages. Although relationship quality can moderate these associations, its effects are limited. These findings underscore the importance of social and familial support in mitigating the negative psychosocial impact of FM, while conflicts or poor‐quality relationships may exacerbate symptom severity.

The majority of participants reported a family income between 1 and 3 minimum wages (69.2%), highlighting that FM disproportionately affects women with lower socioeconomic status. This is supported by a Brazilian study showing that FM is prevalent among adults of low socioeconomic status assisted by the public primary healthcare system, with affected individuals experiencing significant impairments in pain, fatigue, functional ability, and mental health (Assumpção et al. 2009). Similarly, a Danish population‐based study demonstrated that individuals with FM and their spouses had lower employment income, higher healthcare costs, and increased societal burdens compared to matched controls, with many patients experiencing loss of income years before diagnosis and a 50% disability pension rate 10 years after diagnosis (Amris et al. 2024). These findings emphasize the need for accessible, low‐cost interventions such as AP, particularly in public healthcare settings, as a strategy to alleviate symptoms, improve functional capacity, and reduce the socioeconomic impact of FM on patients and their families.

Concerning occupation, housewives and employed women were equally represented (30.8% each), while the predominant education level was high school completion (46.1%). These findings align partially with studies showing that women with FM often have lower health literacy and are more likely to have lower income and educational attainment, factors associated with greater disease impact and limitations in daily activities (Oliveira et al. 2024; Przekop et al. 2010; Büyükşireci and Demirsoy 2021). Lower educational and income levels may reduce access to health information, adherence to interventions, and the perceived benefit from therapeutic exercises, while older age and comorbidities further exacerbate pain‐related restrictions (Przekop et al. 2010; Büyükşireci and Demirsoy 2021). Therefore, understanding occupational and educational profiles is essential to tailor interventions such as AP to optimize outcomes and ensure accessibility for women with FM.

The intervention yielded significant reductions in pain intensity, as measured by the VAS scale, from severe to mild‐to‐moderate. These results are consistent with prior studies indicating that AP and hydrotherapy effectively alleviate pain, enhance functional capacity, and improve quality of life in women with FM (Rodríguez‐Huguet et al. 2024; Silva et al. 2018). Meta‐analyses have demonstrated that AP produces clinically relevant benefits in pain reduction (VAS), functional ability, stiffness, and aerobic capacity, with effects often superior to land‐based exercise or no treatment. Mechanistically, the analgesic effects of AP may be attributed to the buoyancy and thermal properties of water, which reduce mechanical load and joint stress, promote muscle relaxation, and enhance peripheral circulation. Additionally, warm water immersion may stimulate endorphin release, thereby modulating central and peripheral pain pathways and contributing to both physical and psychological improvements in this population (Ma et al. 2024; Lima et al. 2013).

Regarding sleep quality, significant improvements were observed in the global PSQI score (16–9) as well as in the domains of subjective sleep quality, sleep latency, duration, habitual efficiency, and sleep disturbances. Similar findings have consistently shown that AP effectively improves pain, functional capacity, and sleep quality in women with FM. RTCs and systematic reviews report significant benefits on both physical and psychological outcomes, including reductions in fatigue, stiffness, anxiety, and depression, as well as improvements in daily functioning and quality of life (Silv et al. 2012; Calles et al. 2023; Bravo et al. 2024). Specific gains in sleep dimensions such as latency, efficiency, and daytime dysfunction have also been observed, with meta‐analyses showing favorable effects on PSQI scores (mean differences up to −2.05) (Calles et al. 2023; Bravo et al. 2024). Physiologically, these outcomes are supported by the combined effects of buoyancy, warm water immersion, and hydrostatic pressure, which decrease mechanical load, promote muscle relaxation, enhance circulation, and stimulate parasympathetic activity, thereby modulating pain and facilitating more restorative sleep (Silv et al. 2012; Calles et al. 2023; Bravo et al. 2024).

The current study reinforces the role of AP as a feasible, safe, and effective intervention in school clinics and public health settings, showing consistent benefits for women with FM. Beyond reducing pain and improving sleep quality, aquatic interventions have demonstrated positive effects on functional capacity, fatigue, anxiety, and depression, which together may synergistically enhance the overall quality of life. RCTs indicate that AP can produce significant and sustained improvements, including reductions in disability, pain intensity, and impact of FM on daily life (Fonseca et al. 2021; Peng et al. 2022). Although health education programs also provide relevant benefits, evidence suggests that AP may offer broader and more durable outcomes, particularly when targeting multidimensional aspects of FM (Fonseca et al. 2021). These findings highlight that interventions addressing both physical and psychosocial domains are more effective than isolated approaches, underscoring AP as a promising adjunct in the comprehensive management of FM.

This study has limitations that should be considered when interpreting its findings, including the small sample size, short intervention period, and lack of control over potential hormonal factors. Moreover, only women with FM were included, which restricts generalizability to other populations. Despite these limitations, the study has strengths: it is among the first to explore AP in a school clinic setting, using multiple patient‐reported measures to capture both pain and sleep outcomes. These results suggest that AP may offer a feasible and accessible complementary strategy for managing FM in similar healthcare contexts.

In summary, this study provides evidence that AP is a feasible, safe, and well‐tolerated complementary approach intervention for women with FM in school clinics and public health settings. Participation in the program was associated with clinically meaningfull improvements in pain intensity and multiple dimensions of sleep quality, highlighting its potential to enhance overall physical and psychological well‐being. The intervention demonstrated high adherence and absence of adverse events, reinforcing its pragmatic value and real‐world applicability. Although limited by its pre‐post design without a control group and relatively small sample size, the study provides contextually novel evidence from a Brazilian clinical environment, supporting the integration of aquatic programs into multidisciplinary strategies for FM management. Future research with larger, controlled, and more diverse samples is warranted to confirm these findings and to further explore the long‐term benefits of AP, alone or combined with other interventions.

## 5 Implications of Physiotherapy Practice

The findings of this study highlight the potential of AP as a practical and effective intervention for women with FM, particularly in school clinics and public healthcare settings where access to low‐cost and non‐pharmacological treatments is essential. By significantly reducing pain and improving multiple dimensions of sleep quality, AP can help address two of the most disabling symptoms of the condition. Moreover, the intervention showed feasibility and safety, making it suitable for implementation in community‐based programs and multidisciplinary care. Physiotherapists can consider AP not only as a complementary strategy to conventional management but also as a tool to enhance functional capacity, psychological well‐being, and overall quality of life in this population.

## Funding

The authors have nothing to report.

## Ethics Statement

The study protocol was approved by the institution's Ethics Committee through *Plataforma Brasil* (number: 85200524.2.0000.8907) on February 20, 2025, in compliance with the Brazilian National Health Council Resolutions 466/2012 and 510/2016.

## Consent

All participants provided written informed consent prior to enrollment in the study.

## Conflicts of Interest

The authors declare no conflicts of interest.

## Permission to Reproduce Material From Other Sources

The authors have nothing to report.

## Data Availability

The data that support the findings of this study are available from the corresponding author upon reasonable request.
